# Supplementary material for: Levitation and dynamics of bodies in supersaturated fluids
Source: Nat Commun. 2024 May 9;15:3910. doi: 10.1038/s41467-024-47672-z (PMC11082208; doi:10.1038/s41467-024-47672-z)
Supplement: Supplementary file 1 — Supplementary Information [file 41467_2024_47672_MOESM1_ESM.pdf]

Levitation and dynamics of bodies in supersaturated fluids - Supplementary Information

Saverio E. Spagnolie, Samuel Christianson, and Carsen Grote
Nature Communications, 2024

I. Supplementary Methods

Experiments

A simple method to estimate the lifting force

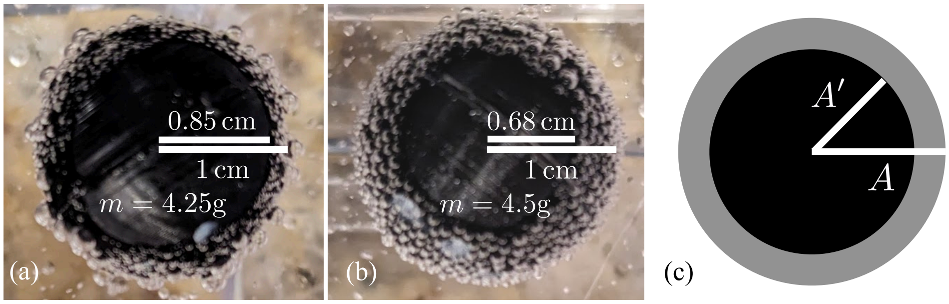

Supplementary Figure 1. **Estimating the lifting force using body emergence.** (a) A lighter body is pushed further out of the fluid by surface bubbles. (b) A heavier body presents less of its surface to the air, and bubble-cleaning rotations become more important. (c) B_s can be reasonably (under-)estimated during the body's brief visit to the surface by measuring the radial distance A' where the body reenters the fluid, as viewed from above.

The saturated surface buoyancy force, B_s, can be estimated by observing the portion of the body which sits outside the fluid while it temporarily resides at the free surface. Figure 1(a,b) show two spherical bodies of radius 1 cm briefly sitting at the fluid-air interface. The lighter body ($m = 4.25$ g) is pushed further out of the water than the heavier body ($m = 4.5$ g). Making the rough approximation that the rest of the surface below has reached its maximal gas capacity, we need only balance the gravitational force with the volumetric and surface buoyancy forces, $mg = \rho g V_S + S'(4\pi A^2)^{-1} B_s$, where V_S is the submerged body volume and S' is the submerged surface area. In terms of the body radius A, and the radial distance of the emerged surface when viewed from above, A' (see Fig. 1c), the top of the body has emerged a distance $h = A\left(1 - \sqrt{1-(A'/A)^2}\right)$ from the surface. With the basic geometric expressions $V_S = (4\pi A^3/3)(1 + h/A)(1 - h/(2A))^2$ and $S' = 4\pi A^2 - 2\pi A h$, we find

$$B_s \approx \frac{2g}{2 - h/A}\left[m - \rho\frac{4\pi A^3}{3}(1 + h/A)\left(1 - \frac{h}{2A}\right)^2\right]. \tag{1}$$

The lighter body in Fig. 1(a), using $A' = 0.85$ cm, produces the estimate $B_s \approx 840$ dyn. Using the heavier body in Fig. 1(b) (which would ideally give a similar estimate), using $A' = 0.68$ cm, we find $B_s \approx 583$ dyn. The latter estimate, taken at approximately $t_0 = 4$ min, is not far from the measured values provided in the main text.

Force development on a 3D-printed sphere at late insertion times

We continued carrying out the (fixed body position) experiment described in the main text to measure the force development on the same 3D-printed spherical body for insertion times longer than an hour. The results, shown in Supplementary Fig. 2 on linear and logarithmic scales, indicate a slowly time-varying power-law force growth for

insertion times close to an hour and beyond. The force growth be behavior is roughly $(t - t_0)^{c(t_0)}$, with $c(t_0)$ a slowly varying exponent which is approximately unity for small t_0 and appears to grow to 2 at larger t_0.

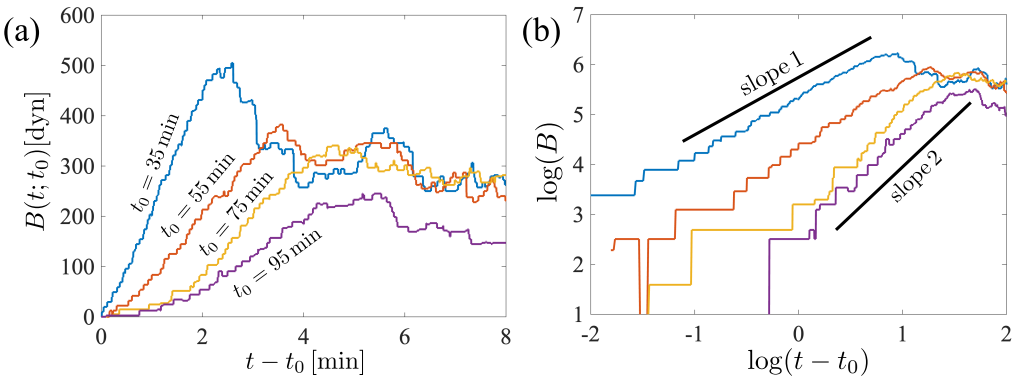

Supplementary Figure 2. **Surface buoyancy force development at later times.** Force on a 3D-printed sphere of radius $A = 1\,\mathrm{cm}$ at late insertion times, on a linear scale (a); and on a log-log scale (b). Power law behavior is observed, $(t - t_0)^{c(t_0)}$, with slowly varying exponent, $c(t_0) \approx 1$ for small t_0, apparently growing to 2 at larger t_0.

Force development on a skewer of raisins

We performed the same (fixed body position) experiment as described in the main text using a skewer of 8 Sunmaid raisins, but using two cans (24 oz) of water in the same container. The results are shown in Supplementary Fig. 3. The skewered 'cylinder' of raisins had approximate length 7cm and diameter 0.8cm. Each raisin was fresh, weighing close to 0.45g; its surface properties appeared to the eye as unchanged over the course of the experiment (significant changes, however, were noted after the raisins were left in the water for 12 hours). To measure the force on the skewer due to bubbles alone, $B(t;t_0)$ shown in Supplementary Fig. 3(a), the weight loss for two cans of water with no immersed bodies was subtracted off. As in the experiment with the 3D-printed body, insertion times began just after depressurizing the fluid $t = t_0 = 0$ and then once again every four minutes.

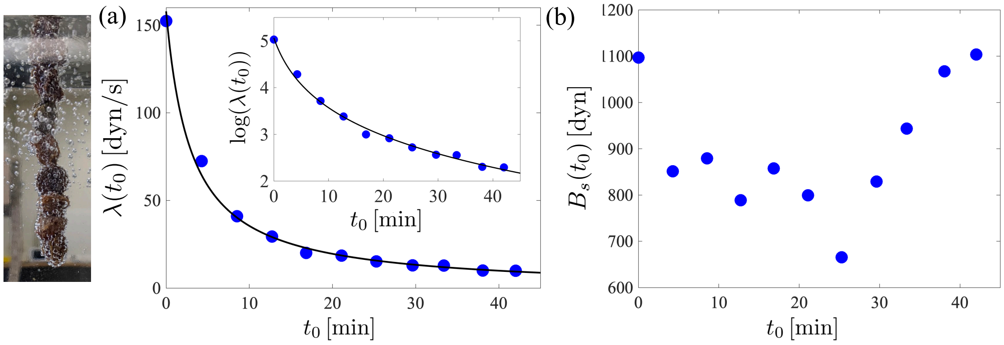

Supplementary Figure 3. **Surface buoyancy growth for eight skewered Sunmaid raisins.** (a) The growth rate for the entire skewer, $\lambda(t_0)$, as a function of the insertion time, t_0, and on a log-linear scale (inset). Curves are a best fit proportional to $S^{3/2}$, as in the main text. (b) The saturated surface buoyancy force, measured 4 minutes after insertion.

With the stabilized surface buoyancy force measured to be roughly 800 dyn, this gives a per-raisin value of $B_s \approx 100$ dyn. An estimated growth rate curve is shown in Supplementary Fig. 3(a) which uses the same form as that described in the main text, but with fitted parameter $\lambda_0 = 19.7$ dyn/min/raisin. Figure 3(b) shows the steady state mean force, measured 4 minutes after insertion. At later insertion times the force growth is slower and smoother, and the stabilized force is non-monotonic in the insertion time, which was observed for some but not all trials using the 3d-printed body as well. This may be due to the cylindrical shape of the raisin skewer, which suffers more dramatically from bubble departure events which may in turn disrupt a greater portion of the exposed body surfaces. A statistical

analysis which includes bubble departure and strong nonlocal interactions may be required to account for this effect. Separately, the bubbles which formed on the raisins, compared to the 3D-printed bodies, were of a rather different character. Due to their rough surfaces, bubbles which formed were fewer and farther between than those observed on the more regular surface of the 3D-printed body, and the lifting force grew over twice as fast.

Mathematical Modeling

Gas escape

When bubbles are rapidly detaching from the container surface, a large-scale circulation emerges, as seen in champagne [1] and beer glasses. We can estimate the gas loss in such a flow as follows. With gas assumed to behave according to an advection-diffusion equation, $c_t + \mathbf{u} \cdot \nabla c = D\nabla^2 c$, with $\mathbf{u}$ the velocity field, the natural boundary conditions are $\mathbf{n} \cdot \nabla c = 0$ on the bottom and side walls of the container, with $\hat{\mathbf{n}}$ the outward-pointing normal vector, and $c(z = 0, t) = 0$ at the top surface, an approximation due to the negligibly small concentration of CO_2 in the air. Regardless of the flow field, the flux of gas from the fluid volume out of the top surface is given by (upon integration by parts and use of the boundary conditions),

$$M'(t) = \frac{d}{dt} \int_{\Omega} c \, dv = D \int_{\partial\Omega} \frac{\partial c}{\partial z} \, dx \, dy. \tag{2}$$

The velocity field enters indirectly but is of critical importance. Consider a flow field which spans the horizontal measure of the container, L, and depth H. We make a coarse approximation that the container supports a single circulation cell, with a single source of bubble production at the bottom container wall, as illustrated in Supplementary Fig. 4. Such a flow includes a stagnation point on the free surface. We approximate the flow as an axisymmetric circulation field of the form $\mathbf{u} = u_r \hat{\mathbf{r}} + u_z \hat{\mathbf{z}}$, where $u_r = (4H)^{-1} L U_b \sin(2\pi r/L) \cos(\pi z/H)$ and $u_z = -U_b[\cos(2\pi r/L)/2 + L \sin(2\pi r/L)/(4\pi r)] \sin(\pi z/H)$. Here $z \in [-H, 0]$, $r \in [0, L/2]$, and U_b is a characteristic velocity scale driven by bubble detachment and rise from the container walls.

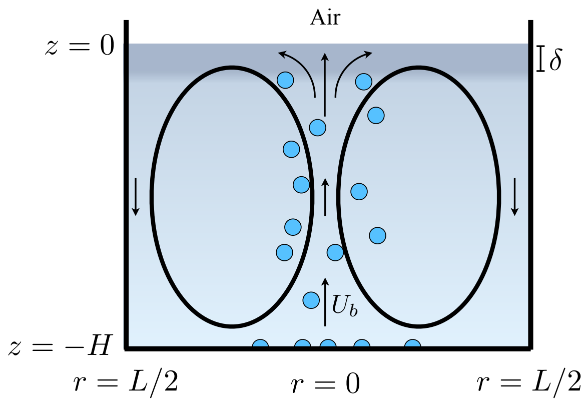

Supplementary Figure 4. **A simple model for estimating gas loss.** A schematic of the large-scale circulation model is shown - a cross-section of a cylindrical container with a single bubble nucleation source at the bottom center. The rate of diffusion out from the free surface (top) is dramatically enhanced by the flow in a boundary layer of thickness δ.

Near the stagnation point the flow has locally the form $\mathbf{u} = (2H)^{-1} \pi U_b(x, y, -2z)$. We now consider the advection-diffusion equation in the inner region where $Z := z/\delta$ is $O(1)$ for some small boundary layer thickness δ. Assuming a steady concentration field, $c_t = 0$, the dominant balance in this inner region is between advection and diffusion, giving $(-\pi U_b/H) z c_z = D c_{zz}$, where taking $c(z = 0) = 0$ and $c(z \to -\infty) = \bar{c}$ gives the solution

$$c(z) = -\text{erf}\left(\left(\frac{\pi U_b}{2DH}\right)^{1/2} z\right) \bar{c}, \tag{3}$$

from which we find $\partial c/\partial z(z = 0) \approx -\bar{c}/\delta$, with $\delta = \sqrt{DH/(2U_b)}$. Writing $M = V_f \bar{c}$, (2) then gives approximately $\dot{\bar{c}} = -\bar{c}/T_r$, where the dot denotes a time derivative and $T_r = \delta V_f/(D S_f)$ is a relaxation time, with S_f the free surface

area and V_f the fluid volume. Using the dimensions of the cubic vessel, $S_f = 79$ cm^2, and $V_f = 355$ cm^3, and an observed free bubble speed of $U_b \approx 1\,$cm/s, the boundary layer is estimated to have thickness $\delta \approx 65\mu$m, and the timescale for gas escape is estimated to be $T_r \approx 26$ min, within range of the relaxation timescale which best fit the data in the main paper. In reality there tend to be multiple points on the surface where bubbles nucleate, and there may be many circulation cells, but accounting for these would appear only to add higher order corrections to the estimate above.

The decay of the supersaturation ratio, $S(t)$, where $S(t) = \bar{c}(t)/c_s - 1$, is desired. Since the velocity field is driven by bubble growth and detachment from the container walls, it diminishes to zero when $S = S_{mc}$, a minimum value associated with the container walls (or remnant particulate matter). We are thus motivated to model this period of gas loss instead by $\dot{S} = -(S - S_{mc})/T_r$, so that further decay of the gas concentration is instead by other means. Namely, after bubbles are no longer forming on and detaching from the container surface, the process tends towards a purely diffusive process, assumed to be governed by approximately one-dimensional dynamics, $c_t = Dc_{zz}$. Taking the container depth H as the linear dimension of interest, the natural timescale from this equation is H^2/D; using experimental values $H = 4.5$ cm and $D = 1.85 \cdot 10^{-5}$ cm^2/s, the timescale of gas loss is on the order of 304 hrs, over 12 days.

The full picture of gas loss involves a change from the mixing performed by large scale circulation to a diffusively-dominated regime. This very rich problem deserves a more substantial investigation.

Discrete and continuum buoyancy growth models

We consider two models of bubble/buoyancy growth, a discrete model and a continuum model, as each can be more appropriate depending on the body size and surface properties. In the discrete model, each of N bubbles are assumed to grow independently according to the bubble growth law attributed to Scriven, based on the Rayleigh-Plesset equation: for an isolated bubble of radius $a(t)$, we have

$$\dot{a} = \frac{D}{a}\left(S - \frac{2\sigma}{pa}\right), \tag{4}$$

where D is a diffusion constant ($D = 1.85 \cdot 10^{-5}$cm^2/s for CO_2 in water [2]), $S(t)$ is the supersaturation ratio, σ is the surface tension of water ($\sigma \approx 70$mN/m $= 70$dyn/cm at room temperature), and p is the pressure near the bubble ($p \approx 1$ atm $= 10^6$ dyn/cm^2) [3–6]. Bubbles smaller than a critical size, $a^* = 2\sigma/pS \approx 1.4\mu$m/S, vanish due to surface tension, while larger bubbles grow, which points to the importance of the length-scale of surface roughness. If $a(0) := a_0 > a^*$, fixing S on the timescale of bubble growth, we have $\dot{a} \approx DS/a$, or $a(t)^2 = a_0^2 + 2DSt$.

We denote by $\mathbf{x}$ a point in space on the body surface, and the body center in the lab frame as $\mathbf{r}(t)$. It is convenient to work in a reference frame of a fixed unit sphere, where the surface is denoted by S_0 with surface points $\mathbf{X}$. We then write $\mathbf{x}(\mathbf{X}, t) = \mathbf{r}(t) + A\mathbf{Q}(t)\mathbf{X}$, where $\mathbf{Q}$ is an orthogonal orientation matrix, with $\mathbf{Q}(0) = \mathbf{I}$. The force conferred by the ith bubble onto the body, with radius $a_i(t)$, is then $\rho g(2\pi/3)a_i^3(t)\hat{\mathbf{z}}$, with ρ the density of water and g the gravitational constant, where we have assumed a hemispherical bubble shape (generally the volume depends on surface roughness and chemistry [7]). The bubbles are assumed to nucleate with a size a_0, based on the length scale of surface roughness, and pinch off above a critical size a_p. The maximum lifting force which can be provided by N bubbles is then

$$B_s = \sum_{i=1}^{N} \frac{2\pi}{3}\rho g a_p^3 = N\frac{2\pi\rho g}{3}a_p^3. \tag{5}$$

Writing the instantaneous lifting force as $B_s F_B[b]\hat{\mathbf{z}}$ (so that $F_B[b]$ is dimensionless), we then have

$$B_s F_B[b] = \sum_{i=1}^{N} \frac{2\pi}{3}\rho g a_i^3, \tag{6}$$

and then

$$F_B[b] = \sum_{i=1}^{N} \left(\frac{a_i}{a_p}\right)^3. \tag{7}$$

The surface buoyancy torque is similarly given by $AB_s\mathbf{L_B}[b] = \sum_{i=1}^{N} \rho g(2\pi/3)a_i^3(t)[(\mathbf{x}_i - \mathbf{r}) \times \hat{\mathbf{z}}]$, where $\mathbf{x}_i$ is the position of the ith bubble in the lab frame. Like $F_B[b]$, $\mathbf{L_B}[b]$ is dimensionless.

The second model considered is a continuum model, more appropriate when the body is covered in a large number of bubbles which are continually growing, merging, and detaching. During this time, the entire body surface presents as a singular large bubble to the surrounding environment. The force growth on the entire surface, if treated uniformly, is given by $\dot{B} = \lambda(t)$, where $\lambda(t) = k(S(t)/S_0)^{3/2}$ for the reasons outlined in the main text. The proportionality constant k is expected to scale with $D^{3/2}$, but also incorporates the more complex behavior of the distribution of bubbles in the neighborhood of each point. With S varying slowly relative to bubble growth, this suggests a linear increase in the total force over time. Later, when S is decaying on a timescale relevant to bubble growth, we expect power-law behavior. This transition is seen in the inset of Supplementary Fig. 2.

Arrival at a steady state value, B_s, is modeled using the modified form $\dot{B} = \lambda(t)(1 - B/B_s)$. In the continuum model, we term the force per area in the lab frame due to bubbles the 'lifting traction', $[B_s/(4\pi A^2)]b(\mathbf{x}, t)\hat{\mathbf{z}}$, where $b(\mathbf{x}, t) \in [0, 1]$ is the proportion of the maximal local lifting contribution, which generally varies over the body, producing both a force and a torque.

II. Supplementary Notes

Glossary

Fluid and container properties

Symbol	Description	Units
δ	Boundary layer thickness	μm
μ	Dynamic viscosity	g(cm s)$^{-1}$
ρ	Fluid density	g/cm^3
σ	Surface tension	dyn/cm
S_{mc}	S value below which bubbles cease to form on the container	dimensionless
H	Fluid depth	cm
L	Container cross-section side length	cm
S_f	Surface area of fluid-air interface	cm^2
V_f	Fluid volume	cm^3
$F(t)$	Scale registered weight of the experimental system at time t	dyn
$M_e(t)$	Total mass loss from the fluid	cm^3

Gas concentration

Symbol	Description	Units
$\bar{c}(t)$	Volume averaged gas concentration	g/cm^3
c_s	Equilibrium gas concentration	g/cm^3
$S(t)$	Supersaturation ratio ($= \bar{c}(t)/c_s - 1$)	dimensionless
k_e	Rate of evaporative mass loss	g/min
D	Diffusion constant for CO_2 in water	cm^2/s

Body geometry and surface properties

Symbol	Description	Units
A	Body radius	cm
m	Body mass	g
ρ_s	(Effective) body density ($= m/V$)	g/cm^3
S_{mb}	S value below which bubbles cease to grow on the body	dimensionless

Body dynamics

Symbol	Description	Units
$\mathbf{r}(t)$	Position of the body centroid	cm
$\mathbf{\Omega}$	Rotation rate	dimensionless
f	Body dancing frequency $(1/\Delta)$	min^{-1}
f_{wobble}	Body wobbling frequency	Hz
C_T	Hydrodynamic drag coefficient	dimensionless
C_R	Hydrodynamic torque coefficient	dimensionless
I_R	Inertial moment coefficient	dimensionless
F_B	Bubble-conferred body force $(= \langle b \rangle)$	dimensionless
$\mathbf{L_B}$	Bubble-conferred body torque	dimensionless
$\mathbf{M}$	Center of surface buoyancy in the body frame	dimensionless
$\mathbf{m}$	Center of surface buoyancy in the lab frame	dimensionless
$\mathbf{Q}$	Orthogonal body orientation matrix	dimensionless
$\mathbf{q}_i$	Orthogonal body orientation basis vectors	dimensionless
V_s	Submerged body volume	cm^3
α	Proportion of submerged body volume $(= V_s/V \in [0,1])$	dimensionless
Re	Reynolds number	dimensionless
W	Vertical velocity	dimensionless

Bubble and lifting force growth

Symbol	Description	Units
$a(t)$	Bubble radius	cm
a_0	Initial bubble radius	cm
a_p	Bubble pinch off radius	cm
$B(t; t_0)$	Surface buoyancy force	dyn
$B_s(t)$	Stabilized surface buoyancy force	dyn
β	Relative lifting force	dimensionless
$b(\mathbf{x}, \text{t})$	Dimensionless surface buoyancy force per area	dimensionless
$\lambda(t)$	Surface buoyancy force growth rate	dyn/s
λ_0	Initial surface buoyancy force growth rate	dyn/s
Λ	Relative bubble growth rate	dimensionless

Times

Symbol	Description	Units
t	Time	min
t_0	Body insertion time	min
t_j	Initial time of the jth (empirical) excursion	min
p_j	End time of the jth (empirical) excursion	min
Δ_j	j^{th} (empirical) excursion time $(= p_j - t_j)$	min
t_{charge}	Surface charging time length	min
s	Dimensionless time	dimensionless
s_0	Dimensionless body insertion time	dimensionless
s_{charge}	Dimensionless charging time length	dimensionless
s_{fun}	Dimensionless insertion time at which s_{charge} is infinite	dimensionless

III. Supplementary References

[1] G. Liger-Belair. The physics behind the fizz in champagne and sparkling wines. *Euro. Phys. J. Special Topics*, 201(1):1–88, 2012.

[2] G. Liger-Belair, E. Prost, M. Parmentier, P. Jeandet, and J.-M. Nuzillard. Diffusion coefficient of CO2 molecules as determined by 13C NMR in various carbonated beverages. *J. Agric. Food Chem.*, 51(26):7560–7563, 2003.

[3] Lord Rayleigh. VIII. On the pressure developed in a liquid during the collapse of a spherical cavity. *Lond. Edinb. Dublin Philos. Mag. J. Sci.*, 34(200):94–98, 1917.

[4] M. S. Plesset. The dynamics of cavitation bubbles. *J. Appl. Mech.*, 16:277–282, 1949.

[5] P. S. Epstein and M. S. Plesset. On the stability of gas bubbles in liquid-gas solutions. *J. Chem. Phys.*, 18(11):1505–1509, 1950.

[6] L. E. Scriven. On the dynamics of phase growth. *Chem. Eng. Sci.*, 10(1-2):1–13, 1959.

[7] L. Pereira, F. B. Wadsworth, J. Vasseur, M. Schmid, S. Thivet, R. B. Nuernberg, and D. B. Dingwell. The physics of dancing peanuts in beer. *Roy. Soc. Open Sci.*, 10(6):230376, 2023.
