# Supplementary material for: Levitation and dynamics of bodies in supersaturated fluids
Source: Nat Commun. 2024 May 9;15:3910. doi: 10.1038/s41467-024-47672-z (PMC11082208; doi:10.1038/s41467-024-47672-z)
Supplement: Supplementary file 3 — Description of Supplementary Files [file 41467_2024_47672_MOESM3_ESM.pdf]

Levitation and dynamics of bodies in supersaturated fluids - Description of Additional Supplementary Files

Saverio E. Spagnolie, Samuel Christianson, and Carsen Grote

Supplementary videos

File Name: Supplementary Movie S1

Description: Sunmaid-brand raisins in a glass container, filled with Klarbrunn-brand carbonated water, perform vertical dancing and wobbling.

File Name: Supplementary Movie S2

Description: Bubble growth and coalescence, and arrival at a fluctuating steady state, on a fixed 3D-printed sphere, at an early insertion time, $t_0 = 1\,\text{min}$. At 10x speed.

File Name: Supplementary Movie S3

Description: Bubble growth and coalescence, and arrival at a fluctuating steady state, on a fixed 3D-printed sphere, at an intermediate insertion time, $t_0 = 10\,\text{min}$. At 10x speed.

File Name: Supplementary Movie S4

Description: For bodies with a dense array of surface bubbles, rotational freedom can be of critical importance for vertical dancing. When the body rotates at the fluid-air interface, the surface is cleared of a larger number of buoyancy-conferring bubbles, and a vertical excursion becomes far more likely.

File Name: Supplementary Movie S5

Description: The motion of nearby bodies can provide a perturbation, nudging the body to rotate and plummet.

File Name: Supplementary Movie S6

Description: The 3D-printed body dancing in carbonated water for two hours, at 4x speed.

File Name: Supplementary Movie S7

Description: Numerical simulations using the discrete bubble model explore the role of the number of bubbles, the dimensionless bubble growth rate, Λ, and the dimensionless maximum lifting force, β.
